# Effect of *N*-glycosylation on horseradish peroxidase structural and dynamical properties

**DOI:** 10.1016/j.csbj.2022.06.008

**Published:** 2022-06-08

**Authors:** Sanja Škulj, Antun Barišić, Natalie Mutter, Oliver Spadiut, Ivan Barišić, Branimir Bertoša

**Affiliations:** a Department of Chemistry, Faculty of Science, University of Zagreb, Horvatovac 102a, Zagreb HR-10000, Croatia; b Molecular Diagnostics, Center for Health and Bioresources, AIT Austrian Institute of Technology GmbH, Giefinggasse 4, Vienna 1210, Austria; c Eko Refugium, Crno Vrelo 2, Slunj 47240, Croatia; d Institute of Chemical Engineering, Research Area Biochemical Engineering, TU Wien, Gumpendorfer Strasse 1a, Vienna 1060, Austria

**Keywords:** *N*-glycosylation, Horseradish peroxidase – HRP, Glycoprotein, Mutations, Molecular dynamics simulations

## Abstract

The effect of different branching types of glycosylation on the structure and dynamics of the horseradish peroxidase (HRP) and an engineered split horseradish peroxidase (sHRP) was studied using all-atom molecular dynamics (MD) simulations. Although tertiary structures of both proteins are stable in the presence, as well as in the absence of glycans, differences in the dynamical properties regarding the presence of glycans were noticed. Fluctuations in the protein structure along both proteins are decreased when glycosylation is introduced. We identified two main regions that are affected the most. The peripheral region is impacted directly by glycans and the central region within the active site with a propagated effect of glycans. Since the mentioned central region in the glycoprotein is not surrounded by glycans and is close to the heme, it is easily approachable to the solvent and substrate. An influence of the glycan presence on the electrostatic potential of the protein and on the heme cofactor was also observed. Altogether, this work presents a global and local analysis of the glycosylation influence on HRP protein’s structural and dynamical properties at a molecular level.

## Graphical abstract

**Figure:**
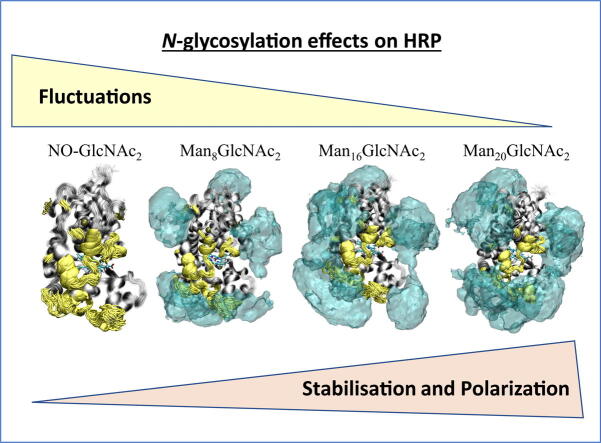

## 1 Introduction

The horseradish peroxidase (HRP) C1A enzyme is an extensively studied peroxidase due to its various potential applications, in biosensors, in immunodetection, and in investigations concerning protein-protein interactions.[1], [2], [3], [4], [5] It catalyzes the oxidation of many electron donor substrates by hydrogen peroxide (H_2_O_2_).[6], [7] Structurally, it consists of 308 amino acids that are mostly alpha folded, a heme cofactor, and two calcium ions (Ca^2+^). Also, its important structural features are four disulfide bridges and nine *N*-glycosylation sites whose role in structural and dynamical properties of HRP are in the focus of the presented research.[8], [9].

Glycosylation, as the most common posttranslational modification of proteins, is biologically important and the influence of glycosylation on structure and stability of numerous glycoproteins has been extensively studied.[1], [10], [11], [12], [13] Lee and coworkers investigated the impact of *N*-glycans on protein structures and dynamics, thereby performing both Protein Data Bank (PDB) structure analysis and molecular dynamics (MD) simulations of glycosylated and deglycosylated proteins.[14] It can be concluded that glycosylation decreases protein dynamics, but does not induce significant changes in the protein conformation.[13], [14] It is also known that glycosylation can stabilize proteins and even affect the active site of proteins.[15], [16] Glycoprotein function is being extensively investigated in new therapies and diagnostics because glycoproteins are targets of pathogens and anomalously glycosylated proteins are important in degenerative diseases.[17].

In the case of HRP, which is in focus of the presented research, it is known that the deglycosylated HRP remains active, with conserved specific activity and reaction kinetics even though showcasing reduced solubility in salt solutions.[18] Further research indicated that non-glycosylated HRP protein expressed in *Escherichia coli* possesses activity.[19] These studies suggest that the HRP protein is active and functional even in the absence of glycans. However, it is assumed that the role of glycans is to maintain the protein conformation, increase protein solubility in water and decrease dynamic fluctuations in protein structure.[1] In favor of this hypothesis is the finding that the unfolding of deglycosylated HRP appears on 2–3 times shorter time scales than the unfolding of native HRP, and that the transition state energy of the deglycosylated HRP is lower compared to native HRP [20] This is confirmed computationally for 63 engineered SH3 domain variants by coarse-grained simulations where thermodynamic stabilization is correlated with the degree of glycosylation and with a glycosylation unfolding barrier increased by approximately 20%.[21] Further, it was found that in a biosensor containing HRP, the glycosylation improves its long-term stabilization.[10] Another example of this phenomenon is the work by Humer and coworkers, where the lack of *N*-glycosylation of HRP resulted in a reduced catalytic activity and thermal stability.[22].

Enzyme engineering is extensively used to improve the activity and stability of glycosylated and non-glycosylated HRP. An example is the recombinant HRP with four glycosylation site mutations (N13D/N57S/N255D/N268D) which has a two-fold higher thermostability and eight-fold increased catalytic activity with ABTS (2,2′-azino-bis(3-ethylbenzothiazoline-6-sulphonic acid)) substrate in comparison to the non-mutated recombinant HRP enzyme.[22] In a recent study, enzyme engineering of an unglycosylated HRP was conducted to improve the stability of unglycosylated HRP C1A for applications in enzyme prodrug cancer therapy.[23] Usually, HRP is isolated from plants and contains 75–80 % of the (Xyl)Man_3_GlcNAc_2_(Fuc) (Xyl–xylose, Man–mannose, GlcNAc–*N*-acetylglucosamine and Fuc–fucose) *N*-glycosylation type with different branching at each *N*-glycosylation site.[24], [25] On the other hand, isolation from yeast *Pichia pastoris* or *Saccharomyces cerevisiae* gives homogeneous *N*-glycans with Man_8_GlcNAc_2_ as a dominant core glycan structure at a volumetric productivity of 70% of the wildtype strain. Heterogeneously hypermannosylation in the Golgi apparatus gives even higher number of mannoses.[26], [27].

Finally, using enzyme engineering and structure-guided cut-site screening followed by two rounds of yeast display evolution enabled the isolation of a stable active form of a glycosylated split horseradish peroxidase (sHRP).[5] This active form consists of two subunits (cut-site is at G213) and six mutations are necessary for the stabilization of the split-form (T21I, P78S, R93G, N175S, N255D, L299R). It has a high potential for applications regarding protein–protein interaction, biosensors, immunoassays, and medical diagnostics. The advantage of using the sHRP is that background signals can be avoided because the two subunits alone lack activity. However, conjunction of the subunits results in the reconstitution of the functional HRP enzyme and recovery of enzymatic activity. Further, since HRP is one of the most sensitive reporter enzymes known,[28] sHRP could be widely applicable for studying mechanisms of communication between a variety of cell types. HRP is well known, while sHRP has not been studied in detail although it presents a new stable active form of a glycosylated split horseradish peroxidase. Since both forms, HRP and sHRP, have large potential for various applications in different areas of research and biotechnology, we were motivated to study both forms, and to compare their properties. The results presented in the paper might contribute to their application in various fields of biotechnology.

Even though the *N*-glycosylation of HRP is extensively studied, there is no detailed insight and explanation of how *N*-glycosylation affects the HRP protein at the atomistic/molecular level. In order to contribute to such explanation, we have performed classical all-atom molecular dynamics (MD) simulations of the HRP. Except the structure of HRP, the engineered split HRP (sHRP) is included to investigate the effect of glycosylation on the mutated split structure concerning intramolecular interactions and to compare it with HRP.[5], [29] The results presented in this paper are focused on the stabilization and structural behavior of the HRP and sHRP proteins induced via *N*-glycosylation since similar effects were noticed for other systems.[14] The final outcomes of the paper confirm the influence that glycans have on the dynamical, electrostatic, and structural properties of the proteins. Therefore, glycans are important, sometimes even necessary, for the proper function of proteins and they must be considered during protein engineering.

## 2 Results and discussion

### 2.1 Effect of *N*-glycosylation on HRP protein structural properties

The effect of glycosylation on the HRP C1A was studied using the native structure (HRP) and the split structure with six mutations (sHRP) introduced by Martell and coworkers.[5] Altogether, eight systems were studied, both proteins were considered in non-glycosylated and glycosylated forms, with three different types of glycan branching, Man_8_GlcNAc_2_, Man_16_GlcNAc_2_ and Man_20_GlcNAc_2_. Since the engineered sHRP is expressed in yeast,[5] we have chosen Man_8_GlcNAc_2_ as a core glycan structure and Man_16_GlcNAc_2_ and Man_20_GlcNAc_2_ glycans types to inspect hypermannosylation effects even though the exact number of mannoses and the branching type on each glycosylation site is not exactly known.[26].

**Fig. 1:**
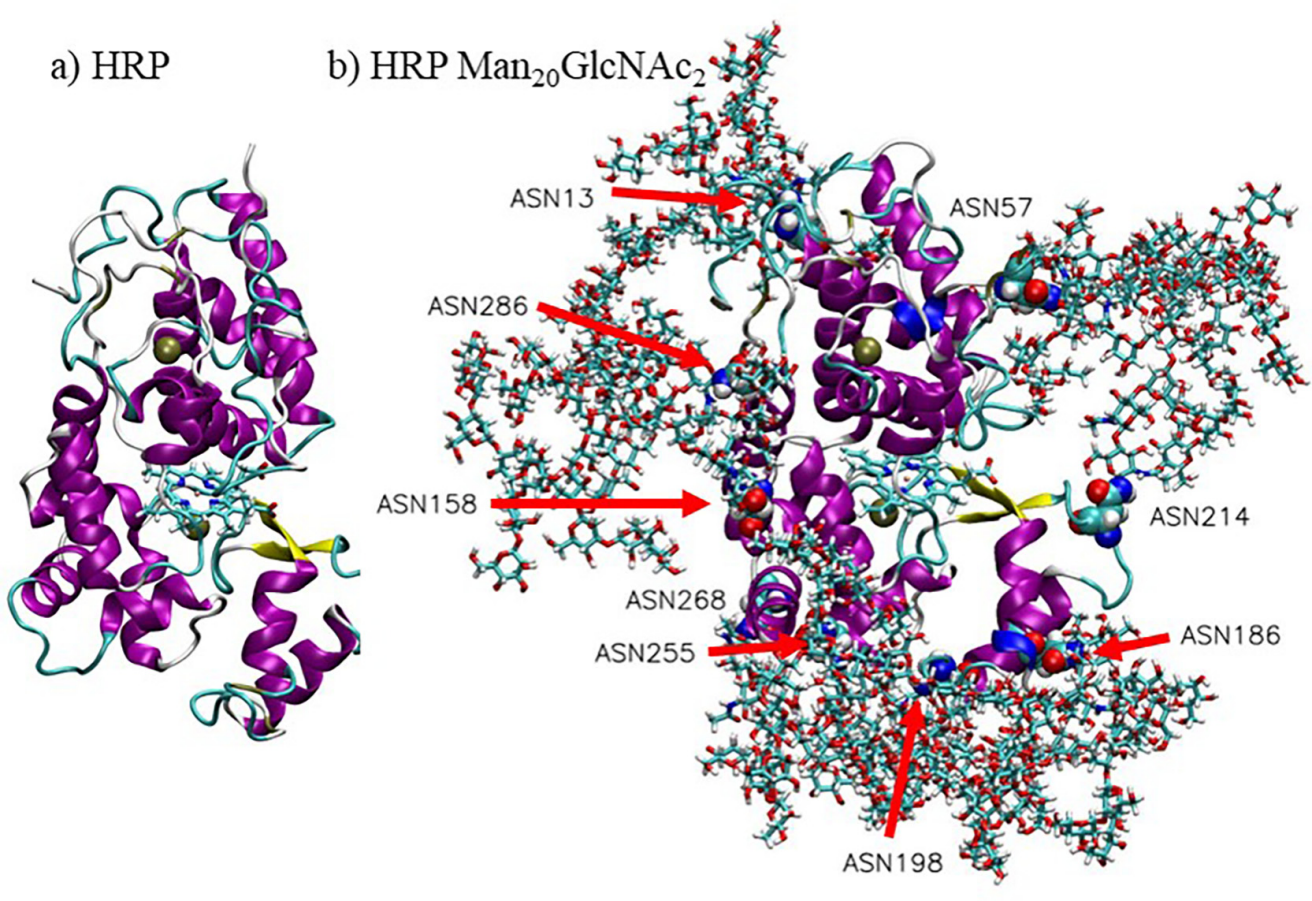
Snapshots of the HRP C1A protein taken at 500 ns of the MD simulation of: a) HRP without glycans and b) HRP with the Man20GlcNAc2 branching type with nine glycosylated asparagine residues indicated with red arrows and shown as VDW spheres. Positions of glycans are the ones found in the structure obtained after 500 ns of MD simulations. (For interpretation of the references to colour in this figure legend, the reader is referred to the web version of this article.)

The most branched glycoprotein Man_20_GlcNAc_2_ is almost one half larger in size measured as a radius of gyration, *R_g_* approximately 2.9 nm comparing to the non-glycosylated protein with *R*_g_ approximately 2.0 nm (Fig. 1 and Table 1). Fluctuations of the radius of gyration and standard deviations increased as glycan branching increased (Fig. S1 and Table1) due to the larger flexibility of glycans with increased branching. (see Section 3 and Video 1 in SI). However, the average value of the protein/glycoprotein radius of gyration, *R*_g_ did not change during the simulations (Table S1 and Fig. S1 in SI). Since the glycoprotein with the glycosylation type Man_20_GlcNAc_2_ is more branched than the other two types of glycosylation, the average radii of gyration of the Man_16_GlcNAc_2_ (approximately 2.8 nm) and Man_20_GlcNAc_2_ (approximately 2.9 nm) glycoproteins are comparable, while for Man_8_GlcNAc_2_ it is smaller with *R*_g_ approximately 2.5 nm (Table 1 and Fig. S1 in SI).

**Table 1:** Averaged values and standard deviations of radius of gyration (*R*_g_) of the (glyco)protein during the MD simulations. If present, glycans were included in *R*_g_ calculations.

System		NO glycan	Man_8_GlcNAc_2_	Man_16_GlcNAc_2_	Man_20_GlcNAc_2_
HRP	*R_g_*/nm	1.98 ± 0.01	2.51 ± 0.03	2.77 ± 0.06	2.89 ± 0.08
sHRP	*R*_g_/nm	1.98 ± 0.01	2.46 ± 0.03	2.75 ± 0.07	2.86 ± 0.06

**Fig. 2:**
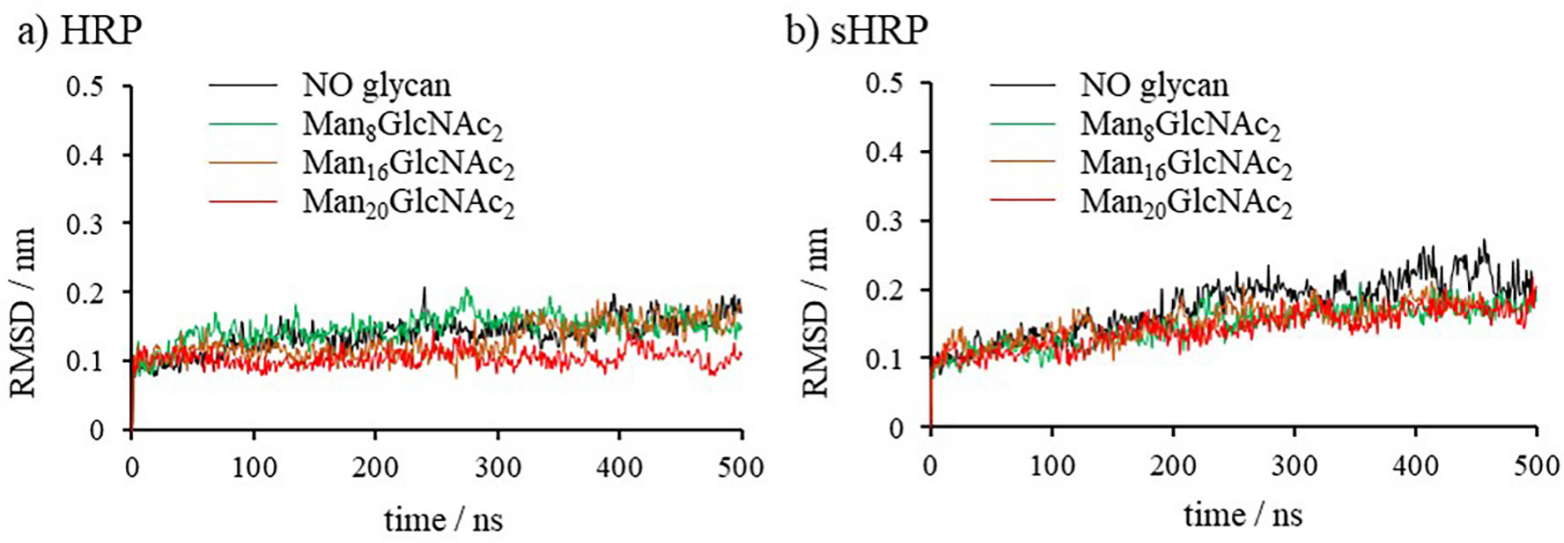
RMSD values of: a) HRP protein and b) sHRP protein with different types of *N*-glycosylation. Backbone carbon atoms – (C_α_) were considered in calculations.

Root mean square deviations (RMSD) of the protein backbone during the simulations show that all systems are equilibrated and stable after 500 ns (Fig. 2) with no significant changes in the overall protein structure (Fig. S2 in SI). In both cases, HRP and sHRP, it was observed that glycan presence did not significantly influence the protein tertiary structure, as well as secondary structure (Fig. S3 in SI) which is in accordance with the literature data on protein structures that *N*-glycosylation does not induce significant changes in protein structure.[14] Slightly larger changes of RMSD in case of sHRP (Fig. 2b and Fig. S2 in SI) comparing to the HRP (Fig. 2a and Fig. S2 in SI) are consequence of two additional C- and N-termini introduced due to the cut-site present only in the sHRP.

**Fig. 3:**
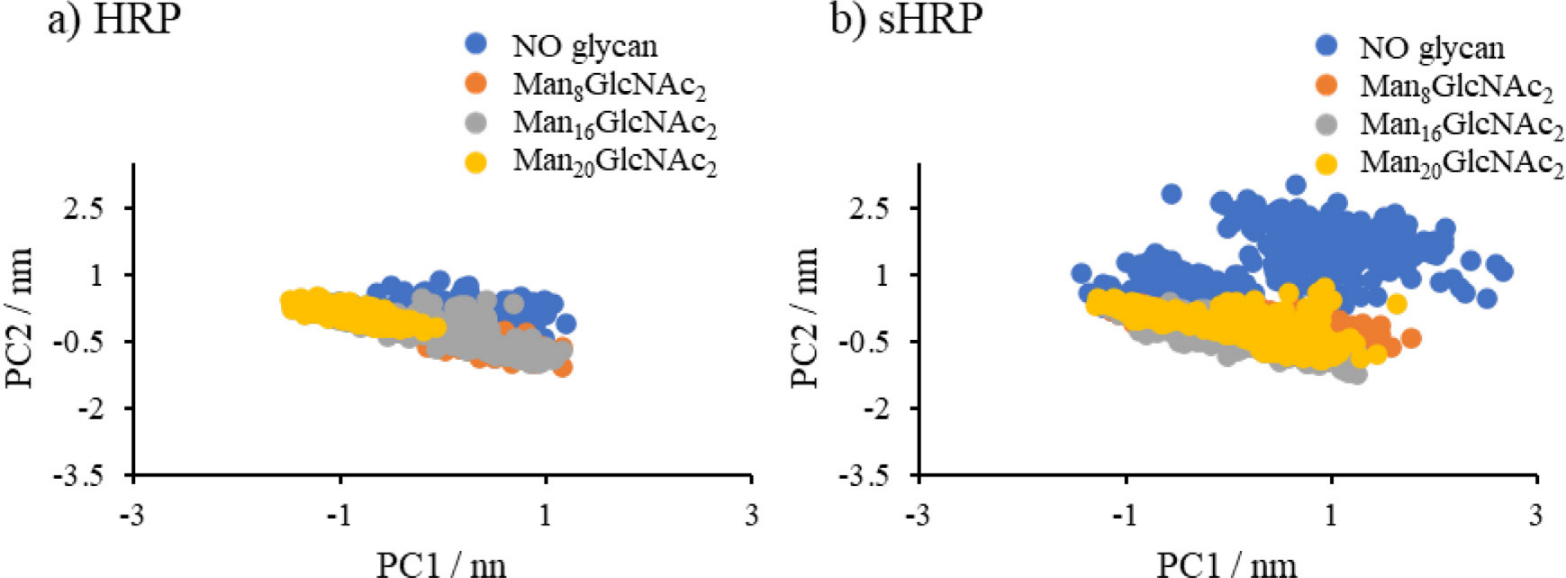
PCA analysis – 2D projection of first two eigenvectors PC1 and PC2 of a) HRP and b) sHRP proteins.

In order to deeply analyze structural properties of the investigated systems, Principal Component Analysis (PCA) of the C_α_ protein backbone atoms during the MD simulations was conducted (Fig. 3). From the movements along the first and second principal components (PC1 and PC2), one can observe that the structure of the HRP remains almost fully preserved, regardless of the presence of glycans. In general, contributions of PC1 for sHRP are higher (13.4.-19.8%) than for HRP (5.5–13.1 %) (Table S3 in SI). On the other hand, the volume occupied by PCA projections in the space spanned by the first two principal components (PC) is smallest in the Man_20_GlcNAc_2_ branching type for both proteins. The comparison of PCA results shows that structural changes are more pronounced for the sHRP (Fig. 3) where the first two PCs span cover a larger area compared to the HRP in all systems. These structural changes of the sHRP are caused by the split in the polypeptide chain between the residues 213 and 214. Due to the introduced split, the sHRP possess two additional fluctuating termini compared to HRP. Therefore, the PCA confirmed the observation that *N*-glycosylation does not have a significant influence on the HRP nor sHRP structural properties.

### 2.2 Glycan structural and dynamical properties

**Fig. 4:**
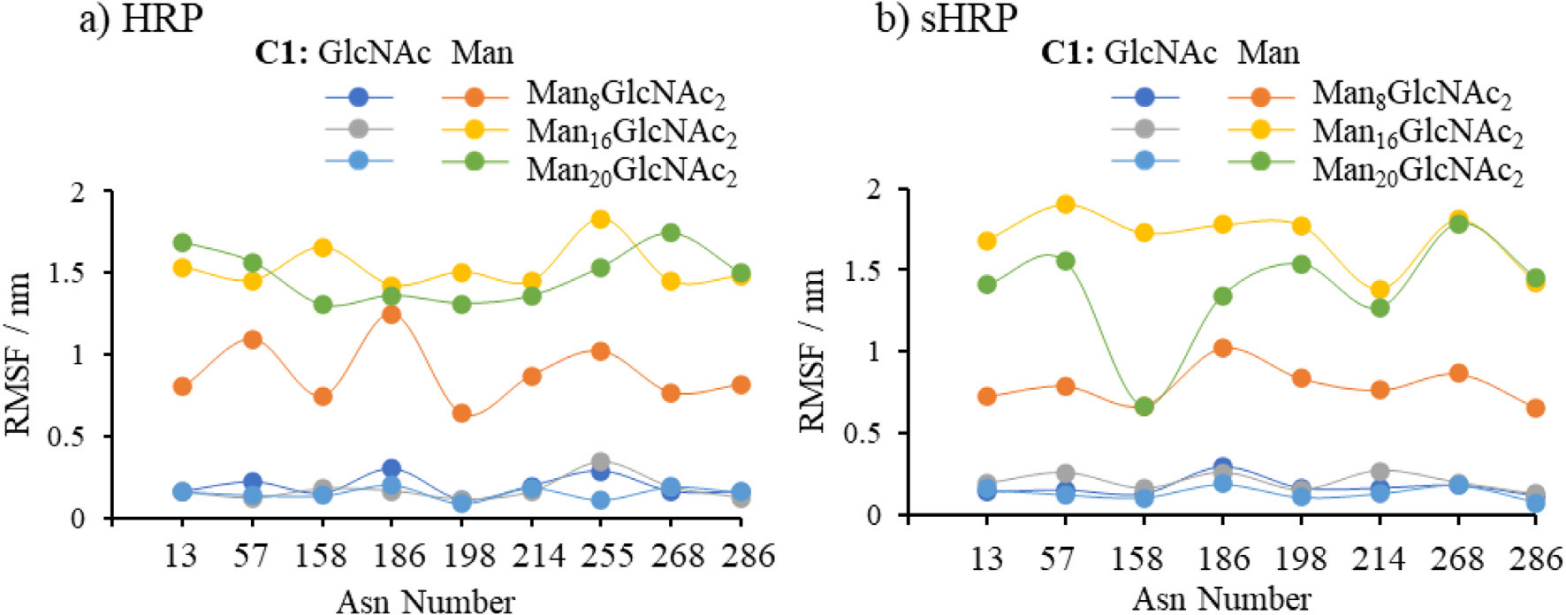
RMSF values of glycans for: a) HRP and b) sHRP protein. The figure presents RMSF of the C1 atom of the protein’s closest *N*-acetylglucosamine (GlcNAc) connected to Asn and corresponding C1 atom of the farthest (Man8, Man16 and Man20) glycan. GlcNAc states for *N*-acetylglucosamine and Man for mannose.

In glycoproteins, glycans occupy a large part of the space around the protein since they are mobile and fluctuate more than the protein itself (Fig. 1b, Fig. 4 and Video 1 in SI). Even the protein’s nearest glycan, GlcNAc, is fluctuating in average more than the protein. Fluctuations of HRP’s and sHRP’s GlcNAc are approximatively 0.2 ± 0.1 nm (Fig. 4, Table S2 in SI). The terminal ends of the protein fluctuate by far the most during the simulations. In average, the oscillation (Table S2 in SI) of the glycoprotein’s Man_20_GlcNAc_2_ (HRP 1.5 nm and sHRP 1.4 nm) and Man_16_GlcNAc_2_ (HRP 1.5 nm and sHRP 1.7 nm) ends are similar, while Man_8_GlcNAc_2_ ends fluctuate less (HRP 0.9 nm and sHRP 0.8 nm). This agrees with previous results that the radius of gyration is similar for Man_20_GlcNAc_2_ and Man_16_GlcNAc_2_ glycoproteins.

**Fig. 5:**
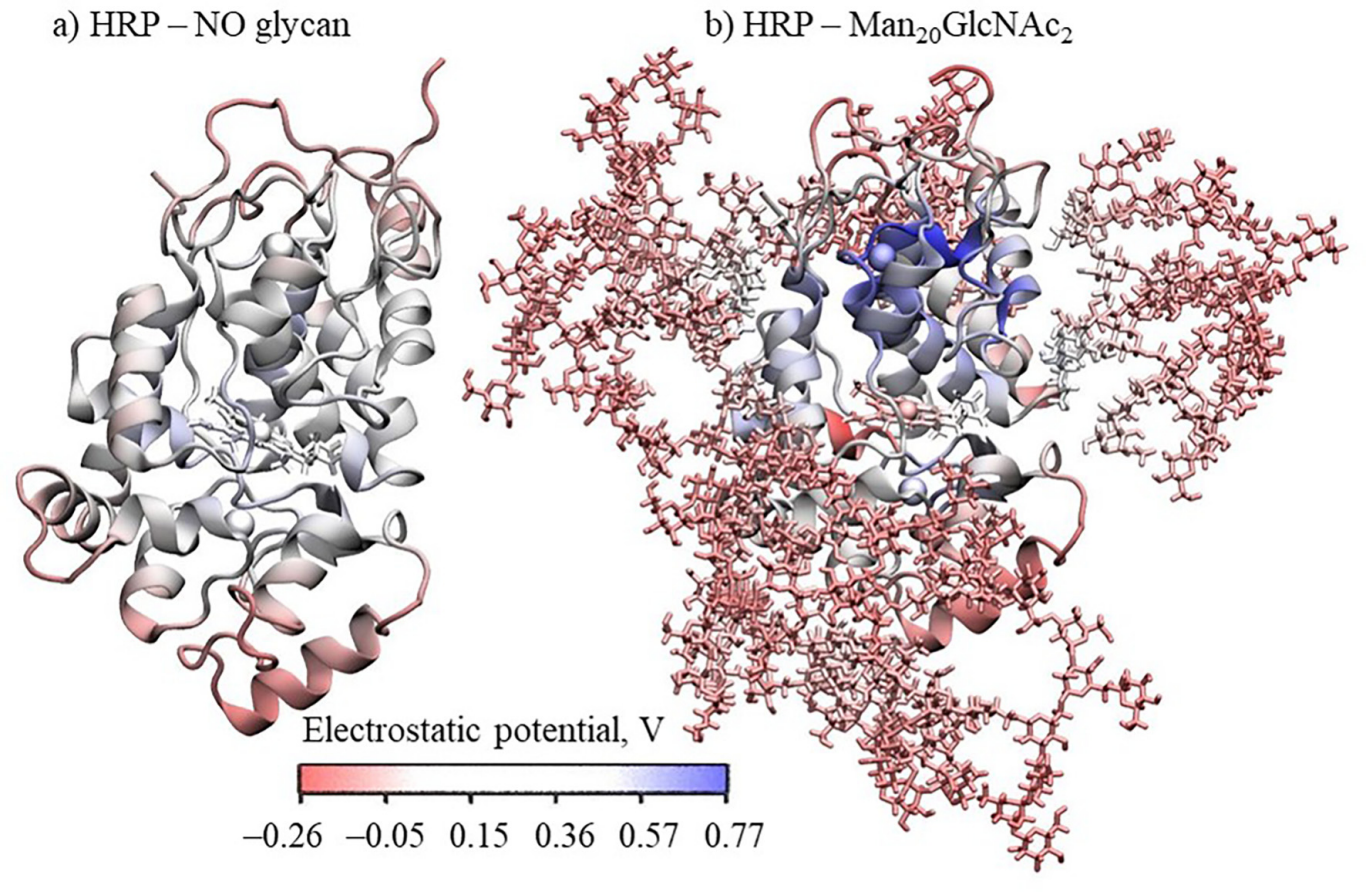
Average electrostatic potential of a molecular dynamic trajectory for: a) HRP, b) HRP with Man_20_GlcNAc_2_ glycosylation. Total variation of potential is 1.03 V.

**Fig. 6:**
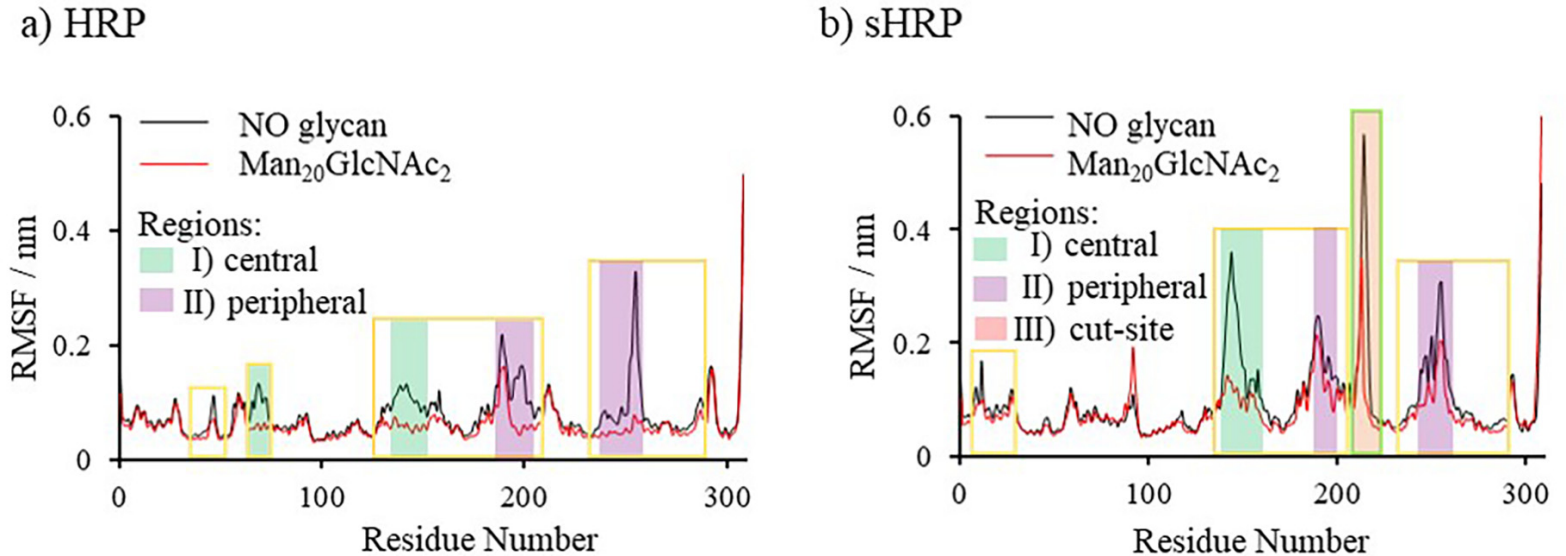
RMSF values of: a) HRP protein and b) sHRP protein. The yellow squares highlight fragments of protein with decreased fluctuations due to glycosylation. The backbone carbon atoms – C_α_ atom of every amino acid was considered in the calculation. Amino acids (fluctuation decreased by > 0.03 nm) close to each other in the tertiary structure forming three regions – I) central, II) peripheral and III) cut-site region are shown in shaded green, purple and red, respectively. (For interpretation of the references to colour in this figure legend, the reader is referred to the web version of this article.)

From the average volume of the glycans around the protein presented in Fig. S4 in SI, it can be concluded that glycans surround some specific surface around the protein’s glycosylated asparagines and “protect” it while the rest of the protein is exposed and more accessible to solvents and substrates. In that way, when glycosylated, every asparagine is locally surrounded by glycans and in that way inaccessible for solvents (Fig. 1). Asparagine fluctuations are mostly decreased when asparagine is glycosylated, especially the cut-site asparagine 214 in sHRP which fluctuations decreased from 0.57 nm in non-glycosylated form to 0.13 nm in Man_20_GlcNAc_2_ glycosylation (Fig. 6). Mainly peripheral sites of the protein are protected by glycans while the central region is exposed directly to solvents (water) and substrates (Fig. S4). This is interesting because in the central region is the heme cofactor which is responsible for the enzyme activity (see next Section 3). As expected, because of the mutated asparagine Asn255Asp in the sHRP, one glycan binding site is missing, and the split enzyme has a smaller glycan volume around the peripheral site than the HRP. Furthermore, the calculated electrostatic potential shows that glycans are slightly more negative than the protein (Fig. 5b). It is also interesting that the presence of glycans induces polarization of the electrostatic potential in the protein and heme cofactor (Fig. 5 and Fig. S5 in SI).

### 2.3 Effect of *N*-glycosylation on HRP protein dynamical properties

The influence of the glycosylation on protein dynamics was studied through fluctuation analysis during the trajectories of the simulated systems (Fig. 6). As expected, the protein has significantly reduced fluctuations compared to the fluctuations of the surrounding glycans (Fig. 4). In general, the glycosylation reduces protein’s fluctuations along the whole length of the protein (Fig. 6, Figs. S6 and S7 in SI). In addition, for sHRP a large effect was observed for the cut-site region. Since the sHRP possess a cut-site between the 213 and 214 residues with two additional C- and N-termini, there is a high peak in fluctuations of the sHRP protein around these amino acids (green square in Fig. 6b) which is not present in HRP (Fig. 6a).

The comparison of HRP and sHRP fluctuations shows the importance of just one glycosylation site difference on protein dynamics. HRP contains nine and sHRP eight glycosylated asparagines since in sHRP Asn255 is mutated to aspartate Asp255 and thus cannot be glycosylated. Due to this, the fluctuations of the non-glycosylated HRP (7.9 ± 3.7) × 10^−2^ nm) are in average by (1.8 ± 3.3) × 10^−2^ nm smaller than of non-glycosylated sHRP (9.7 ± 7.0) × 10^−2^ nm (Table 2).

**Table 2:** Average RMSF (without end residues 300–308) and standard deviation for HRP and sHRP without glycan and with all glycan branching types.

System		NO glycan	Man_8_GlcNAc_2_	Man_16_GlcNAc_2_	Man_20_GlcNAc_2_
HRP	RMSF_av_ × 10^−2^/nm	7.9 ± 3.7	6.8 ± 3.1	7.1 ± 2.9	5.8 ± 2.2
sHRP	RMSF_av_ × 10^−2^/nm	9.7 ± 7.0	7.8 ± 5.0	8.1 ± 4.1	7.6 ± 3.9

**Fig. 7:**
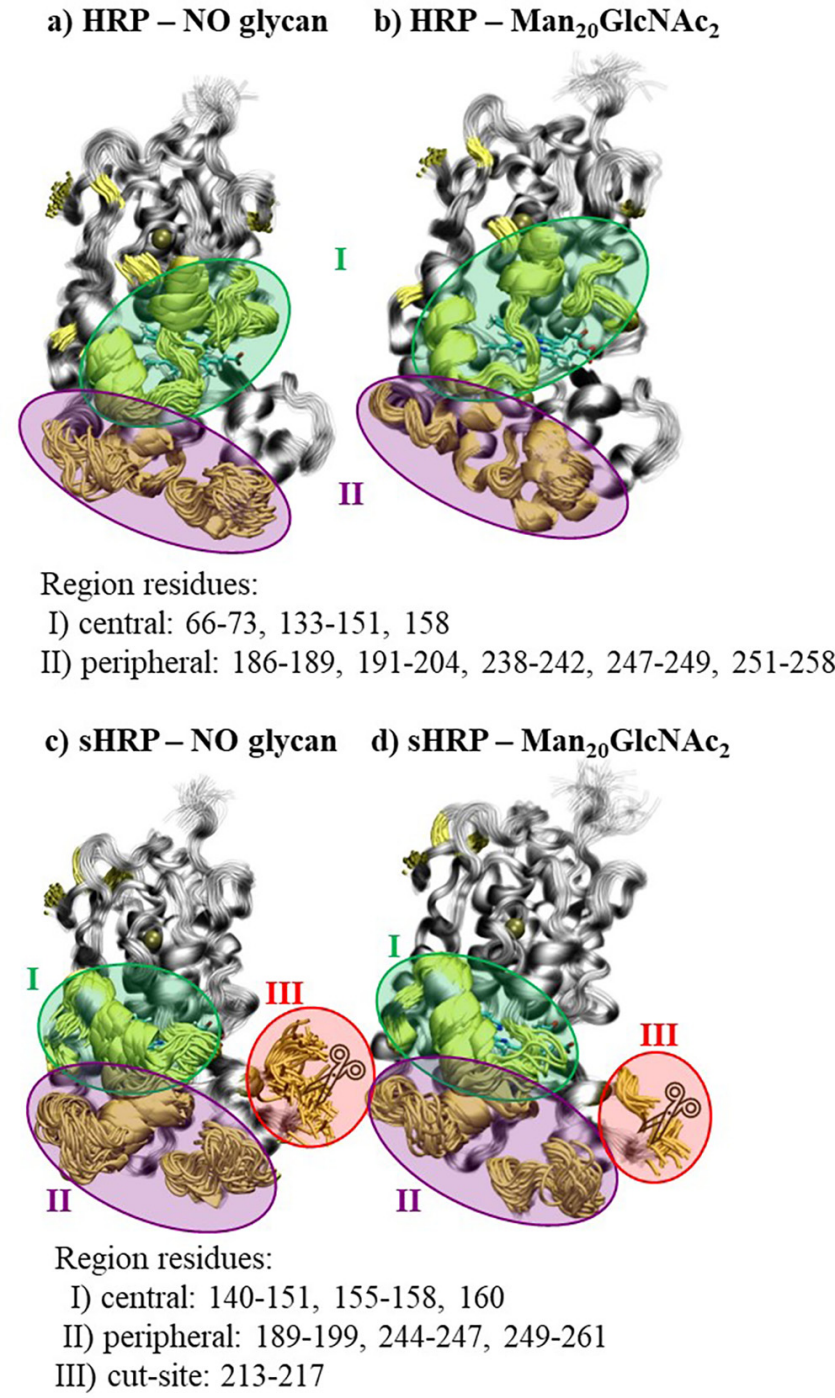
Aligned snapshots of (glyco)protein every 10 ns from trajectory. In yellow are colored residues which fluctuations decreased the most in glycoprotein Man_20_GlcNAc_2_ (fluctuation decreased by > 0.03 nm). Glycans in glycoprotein are omitted for clarity. (For interpretation of the references to colour in this figure legend, the reader is referred to the web version of this article.)

The discussed asparagine (Asn255) is the most fluctuating amino acid during the simulations of the non-glycosylated HRP. When it is glycosylated with the Man_20_GlcNAc_2_ branching type, it has the highest decrease of 0.25 nm (from 0.33 nm for non-glycosylated to 0.07 nm for HRP with Man_20_GlcNAc_2_ branching type). On the other hand, the mutated Asp255 in sHRP with the Man_20_GlcNAc_2_ branching type has a decrease of only 0.11 nm (from 0.31 nm for non-glycosylated to 0.20 nm for non-glycosylated HRP). In the case of the sHRP, beside the C- and N-terminus, the largest decrease of fluctuation (0.23 nm) due to glycosylation with the Man_20_GlcNAc_2_ branching type was observed for Thr144 with fluctuations in the non-glycosylated sHRP of 0.36 nm. The fluctuation differences of the *N*-glycosylated protein are marked in yellow squares in Fig. 6. The structural elements whose fluctuations are the most affected by glycosylation are colored yellow in Fig. 7.

Interestingly, the glycosylation-caused decrease of fluctuations is observed on one part of the protein structure (Fig. S8 in SI, residues 3–32 and 75–129 for HRP, 31–54 and 65–129 for sHRP). The part of the protein structure consisting of residues 3–32 and 75–129 for HRP, 31–54 and 65–129 for sHRP (colored blue on Fig. S8 in SI) is not flexible even without glycans and there are no changes in the flexibility when glycans are present (Fig. 6). Simulations show that different branching types of glycans do not significantly affect fluctuations (Fig. S7 in SI and Table 2). Three main protein regions whose fluctuations are the most decreased due to glycosylation are:

I) central region (HRP amino acids: 66–73, 133–151, 158, sHRP amino acids: 140–151, 155–158, 160; green shaded in Fig. 6 and Fig. 7)
II) peripheral region (HRP amino acids: 186–189, 191–204, 238–242, 247–249, 251–258, sHRP amino acids: 189–199, 244–247, 249–261; purple shaded in Fig. 6 and Fig. 7)
III) cut-site region (sHRP amino acids: 213–217; red shaded in Fig. 6 and Fig. 7).

**Fig. 8:**
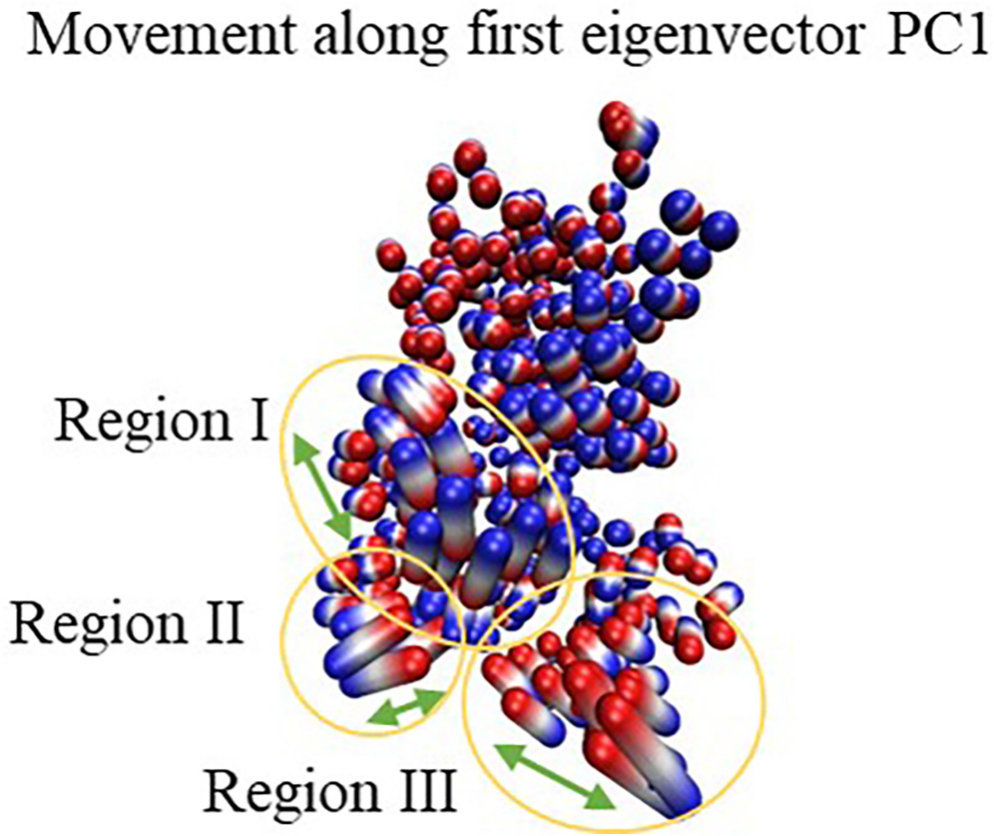
Movements along the first eigenvector PC1 of the protein backbone carbon atoms of all systems together (glycosylated and non-glycosylated, HRP and sHRP). Red and blue are two extreme cases, and white are structures in between. Green arrows illustrate the main direction of the movements along PC1. (For interpretation of the references to colour in this figure legend, the reader is referred to the web version of this article.)

**Fig. 9:**
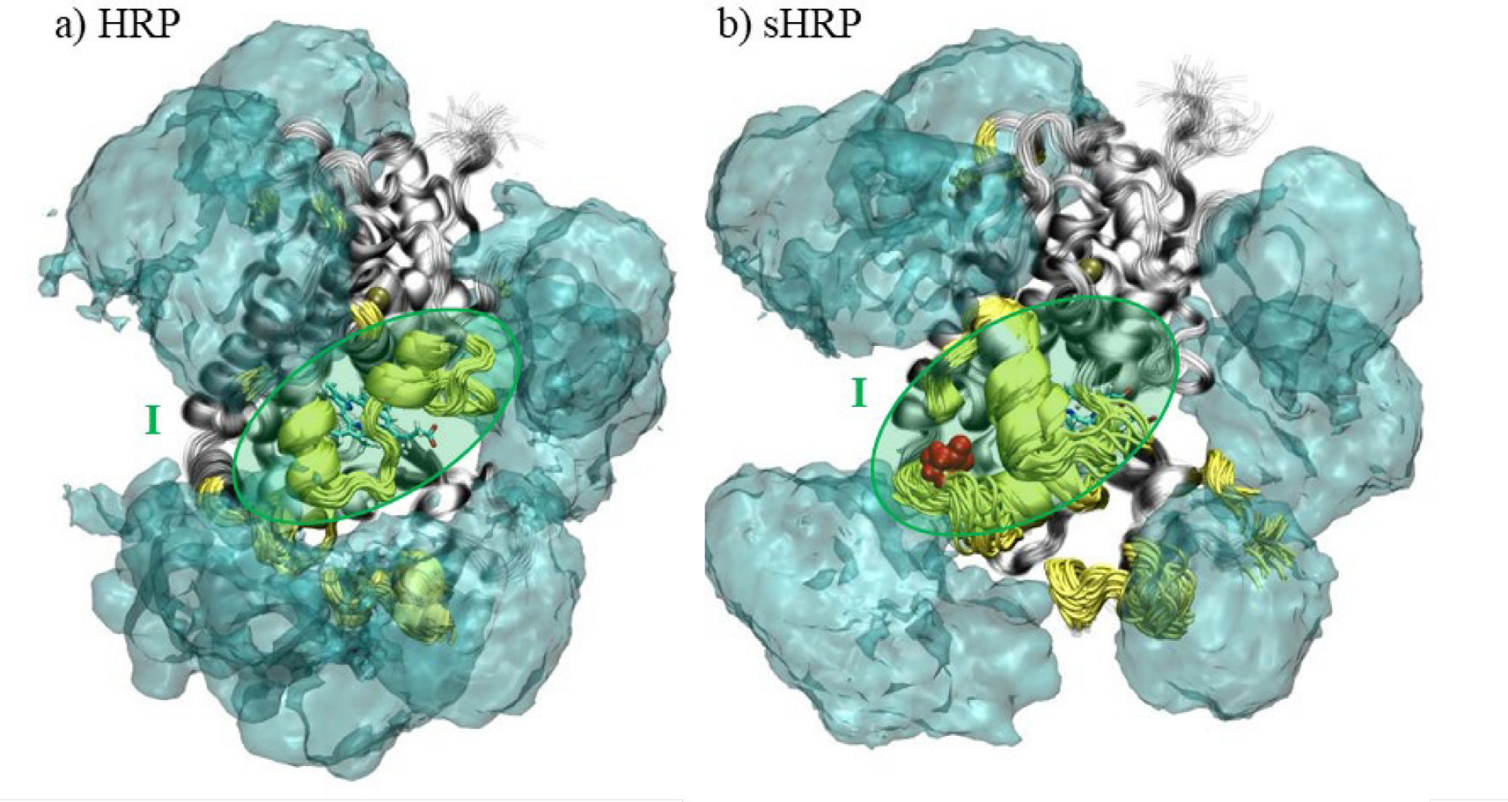
Aligned snapshots of (glyco)protein every 10 ns from trajectory. Yellow colored residues illustrate fluctuations decreasing most in the glycoprotein Man_20_GlcNAc_2_ (fluctuation decreased by > 0.03 nm). The glycans average volume map (isovalue is 0.15) during the 500 ns simulation is presented in cyan. The mutated Asp255 is presented in red – VDW. (For interpretation of the references to colour in this figure legend, the reader is referred to the web version of this article.)

The same regions were identified by PCA, shown in Fig. 8 where main oscillations of both proteins, glycosylated and non-glycosylated, are considered together. Therefore, for HRP and sHRP, the most changeable are three regions, central region I – residues 140–155, peripheral region II – residues 245–255 and cut-site region III – 185–212. These regions, arrived from PC1, correspond to regions determined by RMSF – they only have fewer amino acids included. The central region I is close to the protein core and heme cofactor, it has pronounced fluctuations in the core of the protein (Fig. 9). The same three regions were identified by the PCA as the ones with the most pronounced structural changes along the first eigenvector (PC1) of all simulated systems (Fig. 8 and Fig. S9 in SI). Structural changes of the protein described by the second eigenvector (PC2) are mostly related to the C- and N- terminus, as well as the loops of protein (Fig. S10).

### 2.4 Propagated effect of *N*-glycosylation on HRP protein dynamical properties

Interestingly, the glycosylation influence on protein flexibility is more pronounced for some protein’s regions which are not necessarily in the vicinity of the glycans. Therefore, the highest effect of glycosylation on the protein was observed in two regions, central region I and peripheral region II placed at the proximal site of heme, for both, HRP and sHRP (Fig. 7). The cut-site region III is present only in the sHRP protein. It is interesting that the highest decrease of fluctuations of glycosylated proteins (yellow alpha coils in Fig. 9) is not concentrated only at the protein glycosylation site regions, however it is spread to other areas of the protein, especially to central region I (Fig. 9 and Fig. S4 in SI).

**Fig. 10:**
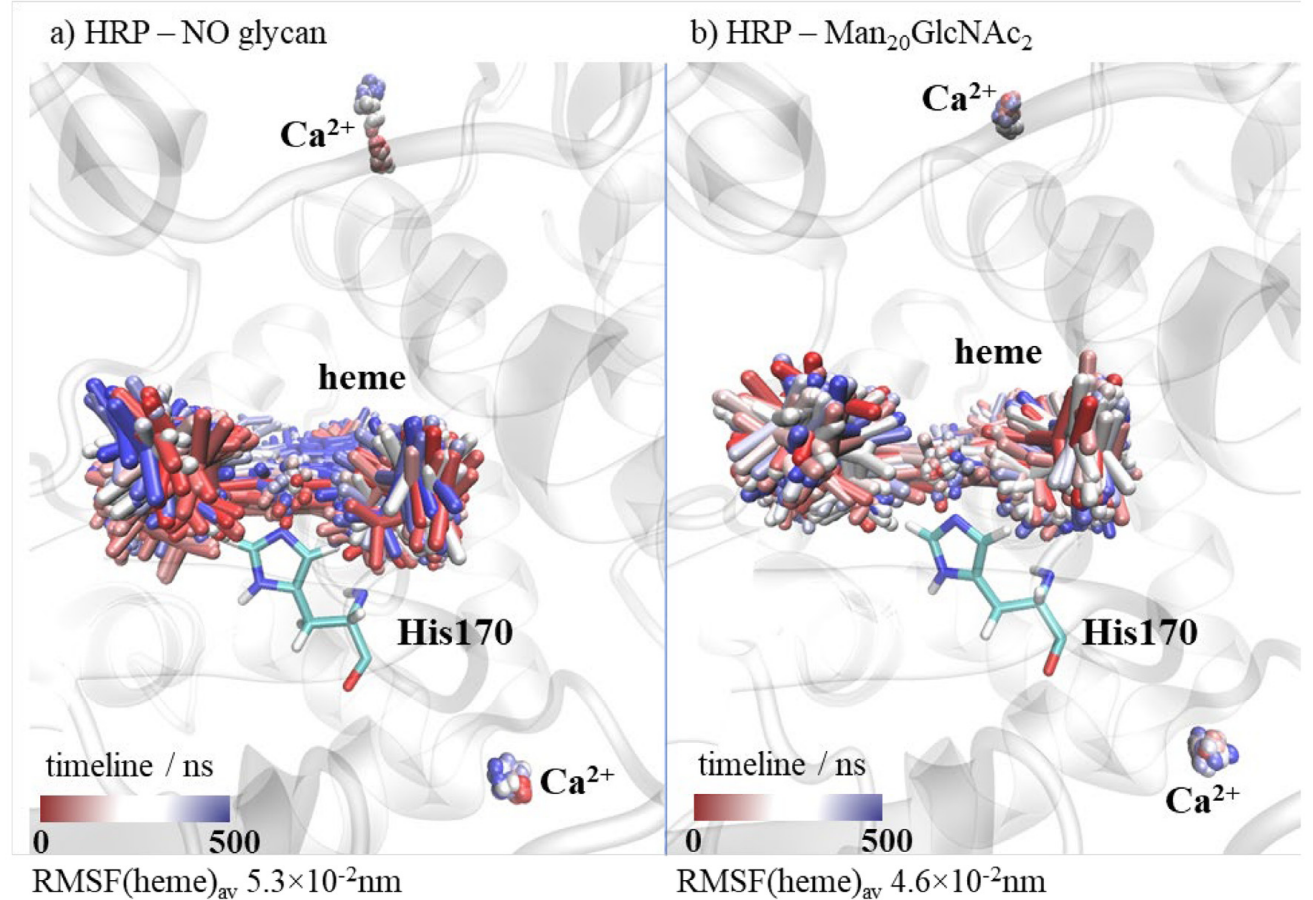
Aligned snapshots of heme cofactor and Ca^2+^ ions every 10 ns from trajectory in time for HRP: a) without glycans and b) with Man_20_GlcNAc_2_ branching type.

This phenomenon is known from literature for different types of glycoproteins and our results support these findings.[14] The peripheral region II is directly affected and protected by glycans, while the central region I is not covered by glycans and is easily accessible for water or other important molecules, such as substrates. The average fluctuations of the central region I amino acid residues during the MD simulations decreased due to glycosylation from (1.1 ± 0.1) × 10^−1^nm in the case of non-glycosylated HRP to (0.6 ± 0.1) × 10^−1^nm for the HRP with the Man_20_GlcNAc_2_ branching type. Even higher changes were noticed for the sHRP where a decrease from (2.1 ± 0.8) × 10^−1^ nm for the non-glycosylated form to (1.1 ± 0.2) × 10^−1^ nm for sHRP with the Man_20_GlcNAc_2_ branching type glycosylation (Table S4 in SI) was observed. This region I is also close to the core of the protein and it is remarkable that the propagated effect of glycosylation is affecting even the core of the protein – the heme cofactor and two calcium ions (Ca^2+^). Since there is the catalytic function, this is the most important location which should be conserved. Oscillations of the heme cofactor and two Ca^2+^ are decreased in HRP with the Man_20_GlcNAc_2_ branching type (from 5.3×10^−2^ nm to 4.6×10^−2^ nm). In non-glycosylated HRP, even the distal calcium ion moved from its position defined in the crystal structure (Fig. 10). In sHRP, this is not the case and the oscillations of both calcium ions are not decreased. It is probably because of the six mutated amino acid residues. Moreover, it is interesting that the electrostatic potential of the HRP and sHRP proteins and the core, including the heme cofactor and Ca^2+^, is changed and more polarized in all types of branched glycoproteins compared to non-glycosylated proteins (Fig. S5 in SI). The heme cofactor has more negative and Ca^2+^ more positive electrostatic potential in glycoproteins compared to non-glycosylated proteins where both potentials are around zero. This polarization of electrostatic potential in glycoproteins is more pronounced in sHRP (Fig. S5e–h in SI).

In summary, the comparison of the HRP with the engineered form (sHRP) pointed to the flexibility as the major difference – sHRP is more flexible. The increased flexibility of sHRP comparing to the HRP is mainly caused by two factors: (i) the presence of the cut-site and (ii) the lack of one glycosylation site. The difference in flexibility is especially pronounced in the case of the non-glycosylated forms. In addition, the glycan presence also increased the polarization of the electrostatic potential of the protein, which is more pronounced in the case of the sHRP. In the end, glycans influence both HRP and sHRP fluctuations, but the tertiary structure of both proteins is conserved even in the absence of glycans. This is in agreement with literature data that the HRP in the absence of glycans is still active and functional,[19] but glycans decrease the dynamic fluctuations of the protein structure[1] and the process of unfolding is 2–3 times faster in the absence of glycans.[20] Glycans oscillate more than the protein and in that way decrease the protein fluctuations. All explanations lead to the conclusion that the overall stability of the HRP and sHRP is increased when glycosylation is present.

## 3 Conclusions

Results of MD simulations of different forms of HRP and sHRP show that *N*-glycosylation does not significantly affect protein tertiary structure, but protein dynamics does change significantly due to *N*-glycosylation. Fluctuations of amino acids are decreased in glycoproteins compared to the non-glycosylated proteins. At the same time, glycans fluctuations are high and they occupy a large space around the protein.

Influence on protein flexibility (fluctuations) is inversely proportional to the glycan size, i.e., protein’s flexibility is more decreased when larger glycans are present. Branching type of *N*-glycosylation does not affect protein’s flexibility. Number of glycosylation sites significantly affects protein’s flexibility. Due to one glycosylation site less (Asn225), decrease of proteins fluctuations are less pronounced in case of sHRP in comparison to HRP.

Decrease in fluctuations was especially observed in central and peripheral region of both proteins, HRP and sHRP. Beside these two regions, in case of sHRP fluctuations of cut-site region is also significantly affected by glycosylation. Interestingly, effect on decrease of fluctuations due to glycans' presence is propagated to the distant central part of protein that contains heme cofactor.

Glycans presence influences the electrostatic potential of HRP. The effect of inducing polarization of electrostatic potential was observed, even for the heme cofactor. Since this region is not surrounded by glycans, it is easily accessible to substrates/water. Therefore, glycosylation provides additional stabilization of HRP and sHRP protein and protection of catalytic heme cofactor.

## 4 Methods

### 4.1 System preparation

**Fig. 11:**
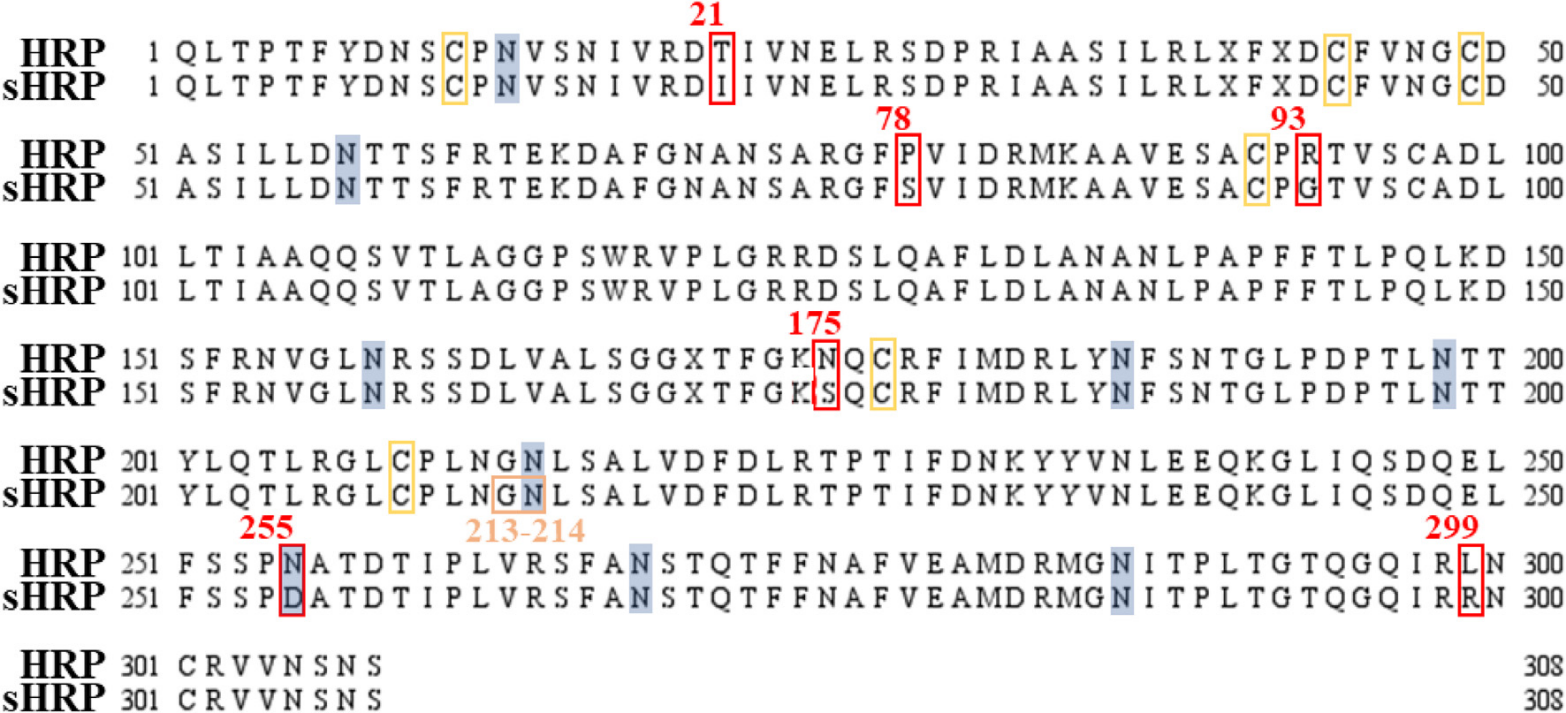
Alignment of HRP and sHRP structure. Positions of mutated amino acids is presented with red square, position of glycosylated sites – asparagine amino acids (which follow pattern Asn–X–Thr/Ser) are shaded in blue square, cysteine which form bridges in yellow squares and cut-site of sHRP is shown in orange square. (For interpretation of the references to colour in this figure legend, the reader is referred to the web version of this article.)

Starting from the crystal structure of horseradish peroxidase C1A from *Armoracia rusticana* (PDB ID: 1H5A), two forms of enzyme were built *in silico*: native (HRP) and split structure with six mutations introduced by Martell and coworkers (sHRP).[5] Main structural motifs, mutation sites, as well as amino acids which form split in sHRP protein are shown in Fig. 11 where primary sequences of HRP and sHRP are aligned.

**Fig. 12:**
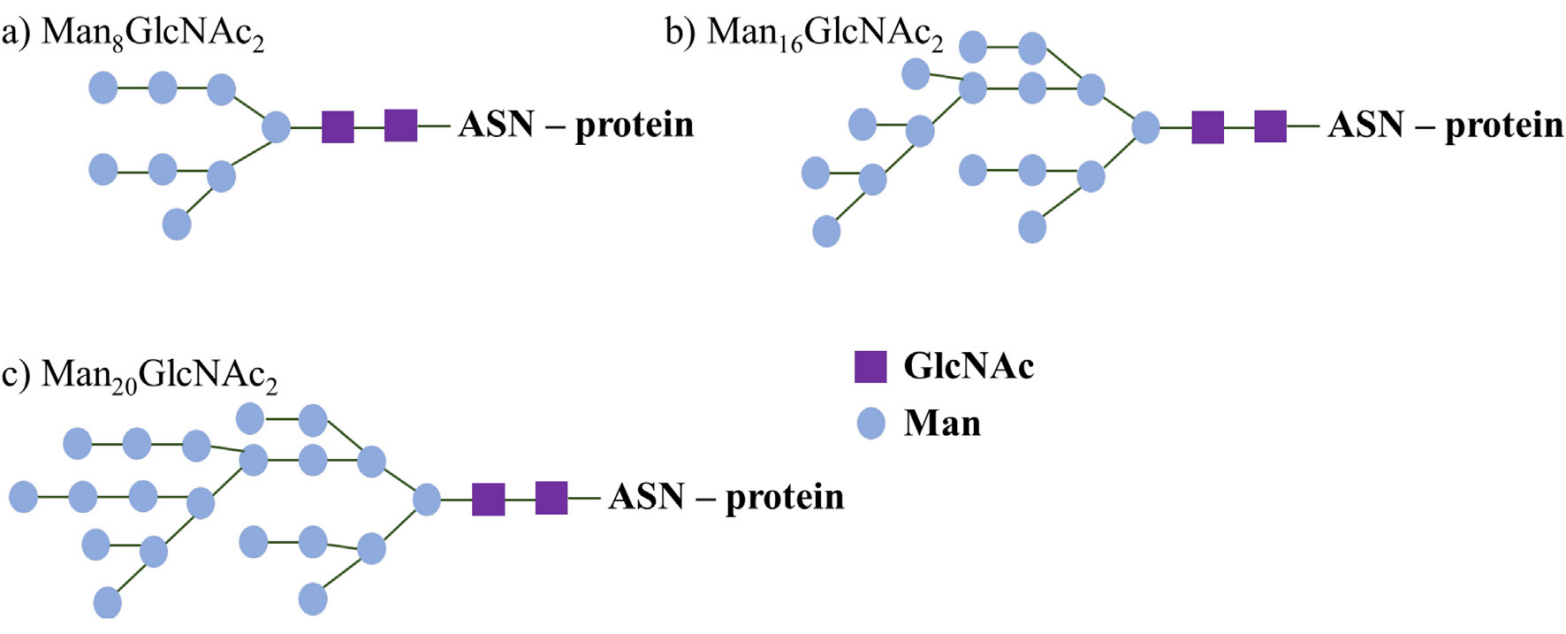
Glycosylation branching types: a) Man_8_GlcNAc_2_, b) Man_16_GlcNAc_2_ and c) Man_20_GlcNAc_2_. Man states for mannose, and GlcNAc for *N*-acetylglucosamine.

Both structures were glycosylated with three different types of glycan. According to number of mannoses, we analyzed systems with three glycosylation branching types: Man_8_GlcNAc_2_, Man_16_GlcNAc_2_, Man_20_GlcNAc_2_ (Fig. 12) and compared it with systems without any glycosylation. It is important to mention that in all model systems every one of nine (HRP)/eight (sHRP) corresponding asparagine amino acids are glycosylated with the same branching type which is not necessary the case in real biological systems. In order to examine *N*-glycosylation effects, structures (HRP and sHRP) were prepared without and with *N*-glycosylation. Further, three different types of glycosylation branching were prepared (Man_8_GlcNAc_2_, Man_16_GlcNAc_2_, Man_20_GlcNAc_2_) as shown in Fig. 12.[26], [30] All asparagine amino acids which follow the pattern Asn–X–Thr/Ser (X is any amino acid residue other than proline and aspartic acid) were *N*-glycosylated – 9 Asn amino acids in HRP (number of Asn residue: 13, 57, 158, 186, 198, 214, 255, 268, 286) and 8 Asn amino acids in mHRP and msHRP (number of Asn residue: 13, 57, 158, 186, 198, 214, 268, 286).

Crystal structure of HRP protein (PDB code: 1H5A, *Armoracia rusticana*)[29] was starting structure for preparing systems that were subjected to molecular dynamics (MD) simulations. Split form (sHRP) was generated from the X-ray structure by introducing the cut-site after G213 and six mutations (T21I, P78S, R93G, N175S, N255D, and L299R) identified by Martell *et al*. Missing residues 307–308 in the X-ray structure were generated by solution builder module of CHARMM-GUI[31], [32], [33] Protonation was achieved using CHARMM-GUI in a way that side chains of all arginines and lysines were positively charged, histidines (with hydrogen on epsilon nitrogen – HIE) and cysteines were in neutral form, while side chains of glutamates and aspartates were deprotonated and negatively charged. Four disulfide bonds (Cys11-Cys91, Cys44-Cys49, Cys177-Cys209, and Cys97-Cys301), as well as bond between His170 and Fe^2+^ from heme cofactor, were added.

CHARMM36m force field was used for parametrization of protein structure, glycans, heme and ions.[34] Solvation effects were simulated using periodic box with at least 20 Å thick layer of TIP3P model of water molecules. Chloride ions were added to neutralize systems. Molecular dynamics (MD) simulations were performed in a periodic boundary condition (PBC) in rectangular box of ∼10 nm × 10 nm × 10 nm in systems without glycans, ∼ 11 nm × 11 nm × 11 nm in systems with glycans Man_8_GlcNAc_2_, ∼ 15 nm × 15 nm × 15 nm in systems with glycans Man_16_GlcNAc_2_ and ∼17 nm × 17 nm × 17 nm in systems with glycan Man_20_GlcNAc_2_. In total, eight systems were prepared for 500 ns of MD simulations (Table 3), four with HRP and four with sHRP in total 4 µs of simulation run.

**Table 3:** Systems prepared for MD simulation.

System	Glycan branching type			
HRP	NO glycan	Man_8_GlcNAc_2_	Man_16_GlcNAc_2_	Man_20_GlcNAc_2_
sHRP	NO glycan	Man_8_GlcNAc_2_	Man_16_GlcNAc_2_	Man_20_GlcNAc_2_

### 4.2 Molecular dynamic (MD) simulations and analysis

Prior to MD simulations, all systems were energy minimized (geometry optimized) in 1000 cycles and then equilibrated in the equilibration process provided by the CHARMM-GUI solution builder module and different time steps and restraints were subsequently applied.[31] After energy minimization, systems were equilibrated for 10 ns. Production phase of molecular dynamics (MD) simulations lasted for 500 ns for each system with a time step of 2 fs and the LINCS algorithm to keep all bonds constrained.[35] MD simulations were performed in the isobaric-isothermal ensemble (NPT) employing periodic boundary conditions (PBC) in all directions at T = 300 K, which was maintained via a Nosé-Hoover thermostat[36] with a coupling constant of 1.0 ps^−1^. Pressure was set to 1.013 bar and was controlled with a semi-isotropic Parrinello-Rahman barostat[37] with a time constant for pressure coupling of 5 ps^−1^. Long rang electrostatics were calculated by the particle-mesh Ewald (PME) method[38] with real space Coulomb interactions cut off at 1.2 nm using a Fourier spacing of 0.12 nm and Verlet cut-off scheme. All simulations were run with the GROMACS 2018.6 software package[39]. Analyses of trajectories were performed using Gromacs tools and VMD program[40]. Secondary structure analysis was performed using Secondary Structure of Proteins (DSSP) tool for the prediction of secondary structure elements from protein.[41], [42].

Electrostatic potential is computed using PMEpot plugin in VMD for each frame and then averaged it over the entire trajectory. Particle mesh Ewald method (PME) algorithm approximates point charges using spherical Gaussians with sharpness controlled by the Ewald factor, electrostatic potential is explicitly calculated solving the Poisson equation over all atoms of the system with three dimensional grid (48×48×48) and Ewald factor of 0.25 at T = 300 K.[43], [44].

## Declaration of Competing Interest

The authors declare that they have no known competing financial interests or personal relationships that could have appeared to influence the work reported in this paper.
