# Supplementary material for: Effect of N-glycosylation on horseradish peroxidase structural and dynamical properties
Source: Comput Struct Biotechnol J. 2022 Jun 8;20:3096–105. doi: 10.1016/j.csbj.2022.06.008 (PMC9233188; doi:10.1016/j.csbj.2022.06.008)
Supplement: Supplementary data 1 [file mmc1.docx]

**Supporting Information**

**Effect of *N*-glycosylation on Horseradish Peroxidase Structural and Dynamical Properties**

Sanja Škulj,^1^ Antun Barišić,^1^ Natalie Mutter^2^, Oliver Spadiut^3^, Ivan Barišić^2^, Branimir Bertoša*

^1^ Department of Chemistry, Faculty of Science, University of Zagreb, Horvatovac 102a, HR-10000 Zagreb, Croatia.

^2^ Molecular Diagnostics, Center for Health and Bioresources, AIT Austrian Institute of Technology GmbH, Giefinggasse 4, 1210 Vienna, Austria

^3^ Institute of Chemical Engineering, research area Biochemical Engineering, TU Wien, Gumpendorfer Strasse 1a, 1060 Vienna, Austria.

* Corresponding author: Dr. Branimir Bertoša

Department of Chemistry, Faculty of Science, University of Zagreb, Horvatovac 102a, HR-10000 Zagreb, Croatia

Phone: +385 1 4606 132

e-mail: bbertosa@chem.pmf.hr

**Table of contents:**

**Page S-2**: Figure S1, Table S1

**Page S-3**: Figure S2, Table S2

**Page S-4**: Figure S3

**Page S-5**: Figure S4

**Page S-6**: Figure S5

**Page S-7**: Figure S6, Figure S7

**Page S-8**: Figure S8

**Page S-9**: Figure S9**Page S-10**: Figure S10, Table S3

**Page S-11:** Table S4


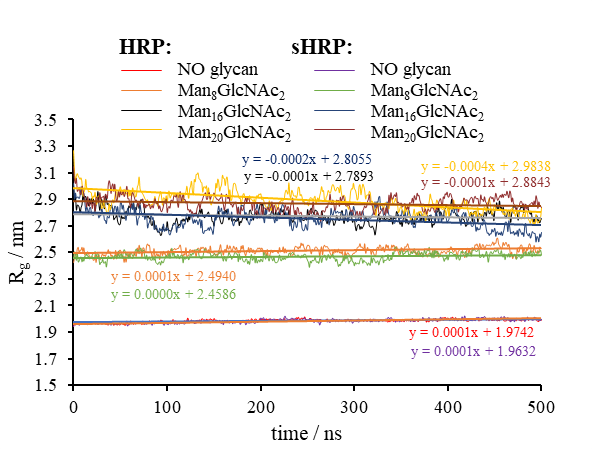


Figure S1. Radius of gyration (*R_g_*) and trendlines with linear equation of all eight systems in nm. If present, glycans were included in *R*_g_ calculations.

Table S1: Averaged values of radius of gyration (*R*_g_) every 100 ns of the proteins during the MD simulations. If present, glycans were included in *R*_g_ calculations.

| **HRP** | **NO glycan** | **Man_8_GlcNAc_2_** | | **Man_16_GlcNAc_2_** | | **Man_20_GlcNAc_2_** | |  |
| --- | --- | --- | --- | --- | --- | --- | --- | --- |
| R_g_ / nm 0-100 ns | 1.97±0.01 | | 2.49±0.03 | | 2.81±0.08 | | 2.93±0.07 | |
| R_g_ / nm 100-200 ns | 1.99±0.01 | | 2.52±0.02 | | 2.76±0.04 | | 2.96±0.06 | |
| R_g_ / nm 200-300 ns | 1.99±0.01 | | 2.51±0.03 | | 2.77±0.03 | | 2.92±0.05 | |
| R_g_ / nm 300-400 ns | 1.99±0.01 | | 2.51±0.03 | | 2.74±0.04 | | 2.85±0.06 | |
| R_g_ / nm 400-500 ns | 1.99±0.01 | | 2.53±0.03 | | 2.79±0.04 | | 2.81±0.04 | |
| **sHRP** | **NO glycan** | | **Man_8_GlcNAc_2_** | | **Man_16_GlcNAc_2_** | | **Man_20_GlcNAc_2_** | |
| R_g_ / nm 0-100 ns | 1.96±0.01 | | 2.47±0.03 | | 2.80±0.08 | | 2.91±0.06 | |
| R_g_ / nm 100-200 ns | 1.97±0.01 | | 2.47±0.03 | | 2.75±0.06 | | 2.87±0.05 | |
| R_g_ / nm 200-300 ns | 1.99±0.01 | | 2.45±0.03 | | 2.77±0.05 | | 2.82±0.04 | |
| R_g_ / nm 300-400 ns | 1.99±0.01 | | 2.47±0.04 | | 2.78±0.04 | | 2.85±0.05 | |
| R_g_ / nm 400-500 ns | 2.00±0.01 | | 2.49±0.02 | | 2.69±0.06 | | 2.88±0.04 | |


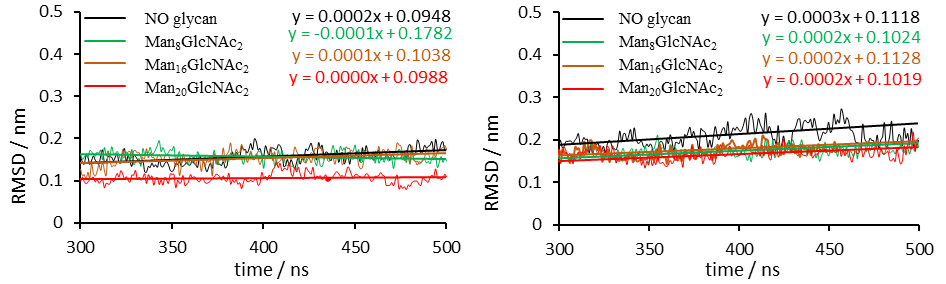


Figure S2: RMSD values and trendlines with linear equation for last 200 ns of MD simulation.

Table S2: Average RMSF and standard deviation for C1 atom of the protein’s closest *N*-acetylglucosamine (GlcNAc) connected to Asn and corresponding C1 atom of the farthest (Man8, Man16 and Man20) glycan in HRP and sHRP. GlcNAc states for *N*-acetylglucosamine and Man for mannose.

| System | | **Man_8_GlcNAc_2_** | | | **Man_16_GlcNAc_2_** | | | **Man_20_GlcNAc_2_** | | |
| --- | --- | --- | --- | --- | --- | --- | --- | --- | --- | --- |
|  |  | GlcNAc | | Man8 | GlcNAc | | Man16 | GlcNAc | | Man20 |
| HRP | RMSF_av_×10^-2^ / nm | 19.6±6.3 | 89.1±19.3 | | 17.4±7.2 | 153.2±13.2 | | 15.4±3.8 | 148.5±16.3 | |
| sHRP | RMSF_av_×10^-2^ / nm | 16.9±5.5 | 79.2±12.0 | | 20.4±5.4 | 168.4±18.6 | | 13.6±4.1 | 137.8±32.7 | |


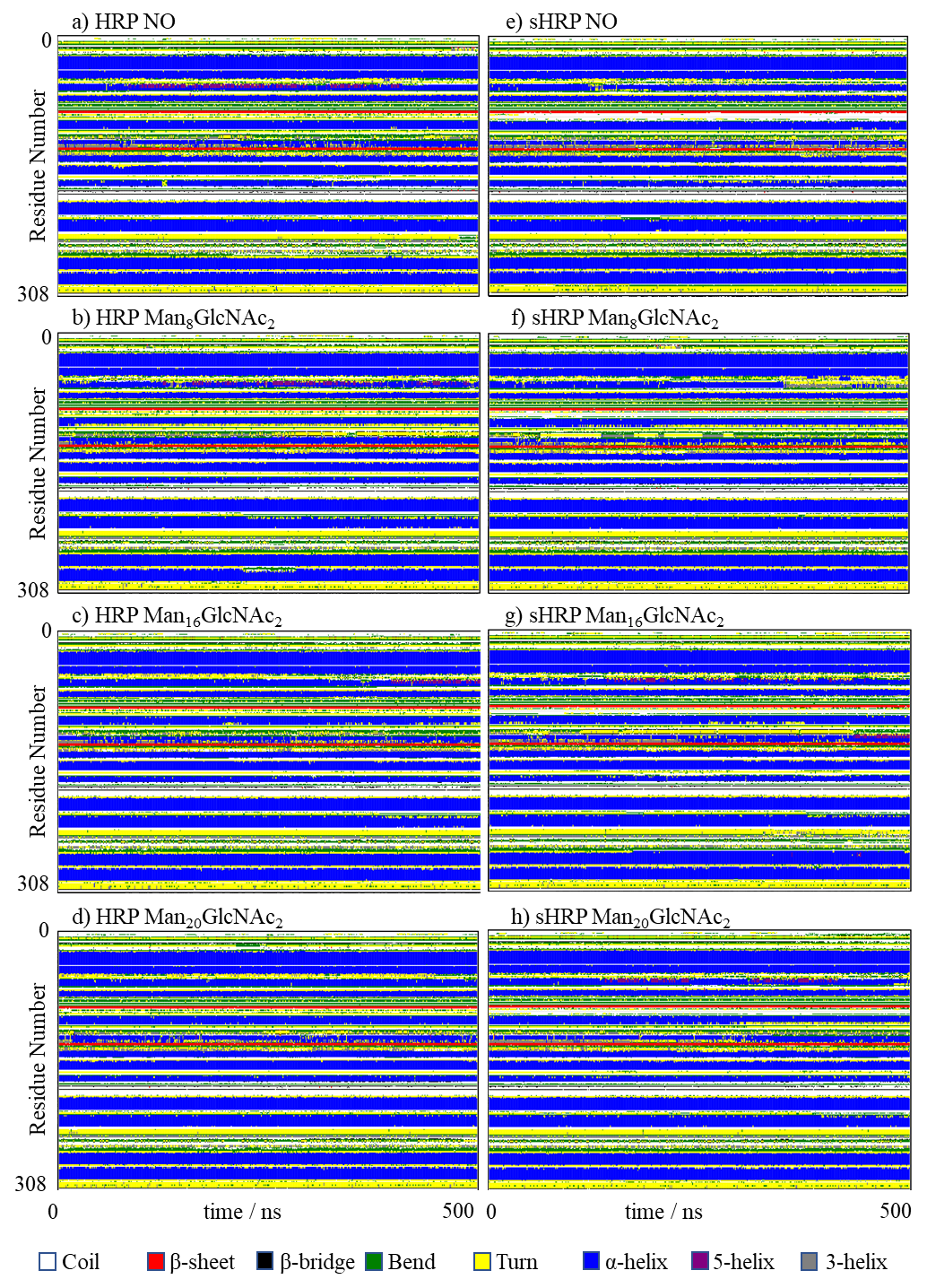


Figure S3. Secondary structure evolution of the HRP and sHRP structures without and with glycans with Man_20_GlcNAc_2_, Man_16_GlcNAc_2_ and Man_8_GlcNAc_2_ branching type. Secondary structure of HRP and sHRP is preserved in time for all systems.


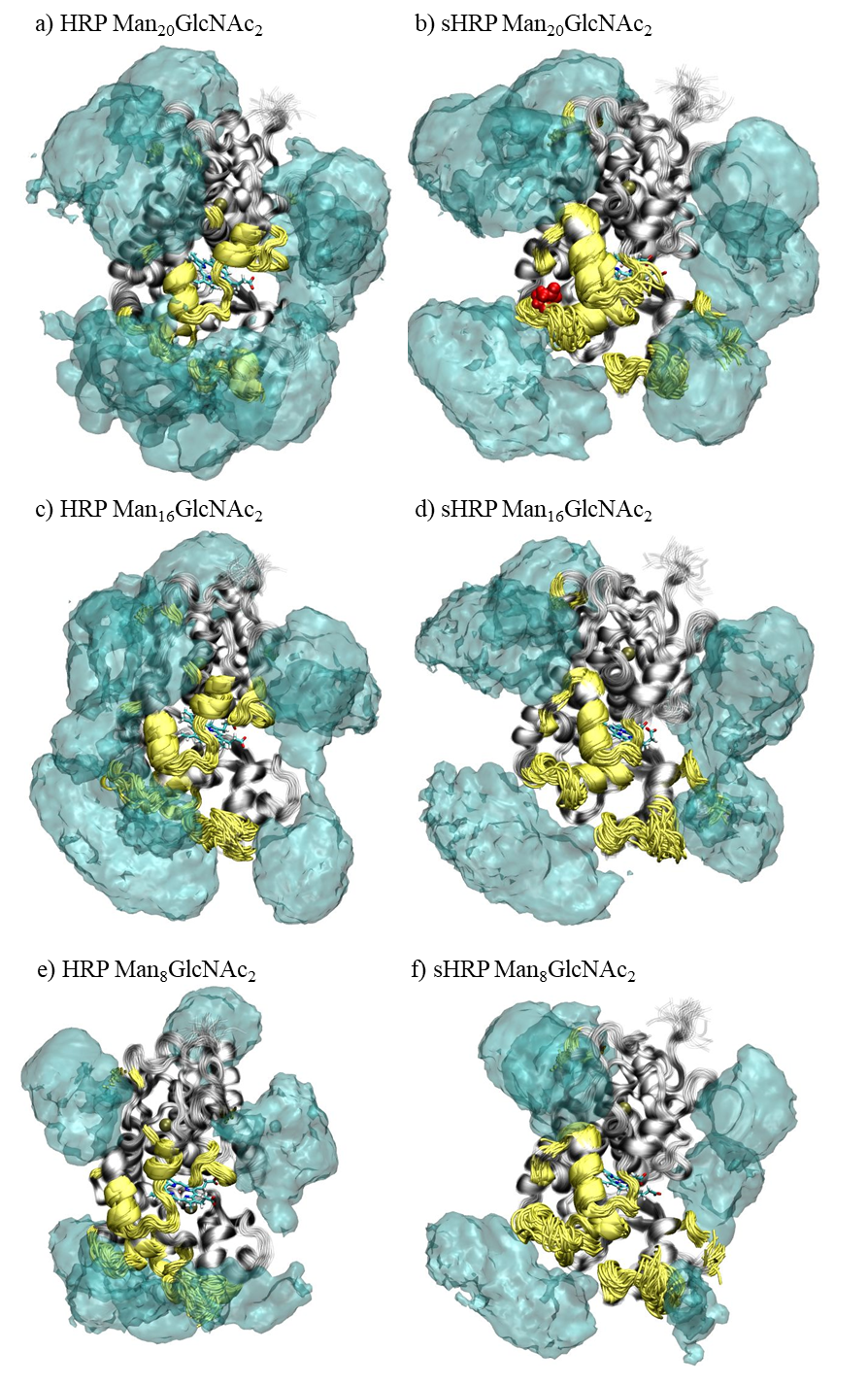


Figure S4. Aligned snapshots of (glyco)protein every 10 ns from trajectory. In yellow are colored residues which fluctuations decreased the most in glycoproteins: Man_20_GlcNAc_2_, Man_16_GlcNAc_2_ and Man_8_GlcNAc_2_ (fluctuation decreased by > 0.03 nm in Man_20_GlcNAc_2_). Glycans average volume map (isovalue is 0.15) during 500 ns simulation is presented in cyan. Mutated Asp255 is presented in red VDW model on Figure b.


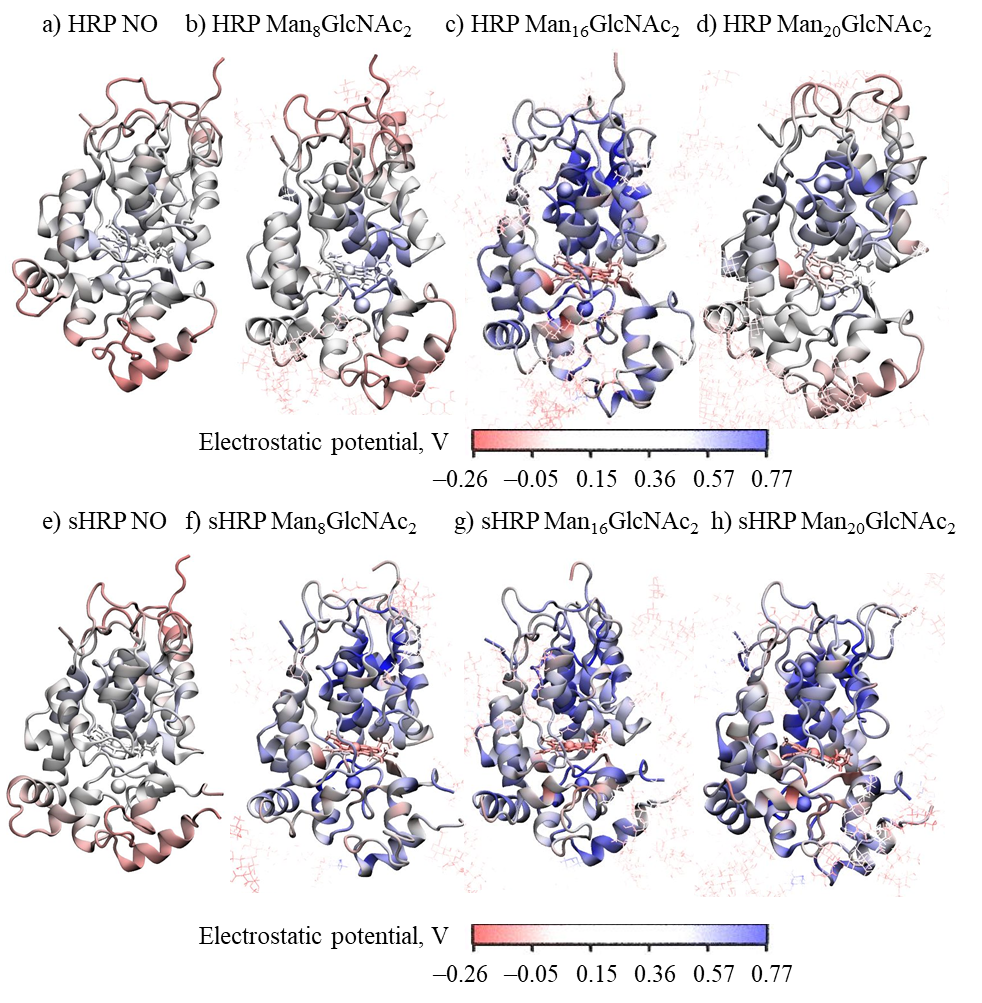


Figure S5. Average electrostatic potential along a molecular dynamic trajectory of different systems. Total variation of potential is 1.03 V.


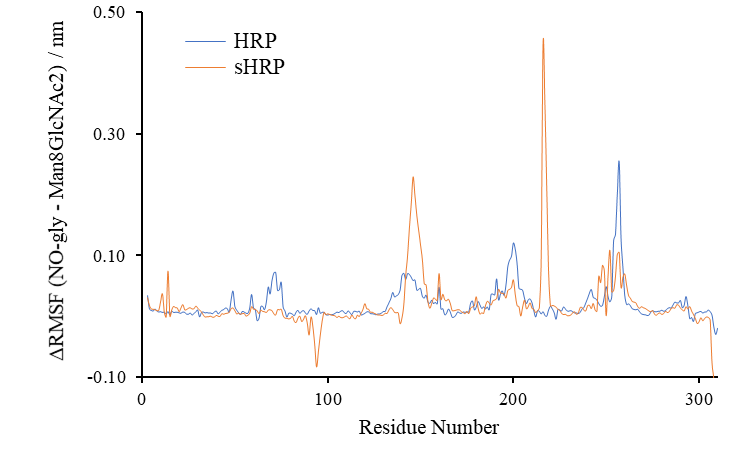


Figure S6. Subtraction of values of fluctuations of protein without glycan and protein with Man_20_GlcNAc_2_ type of branching for HRP and sHRP. In HRP, fluctuations are always decreased with glycoprotein Man_20_GlcNAc_2_ (except residues 295, 306-308 when it is slightly increased). In sHRP, fluctuations are almost always decreased, except in residues 80-81, 84, 87-88, 90-95, 137 and 304-308.


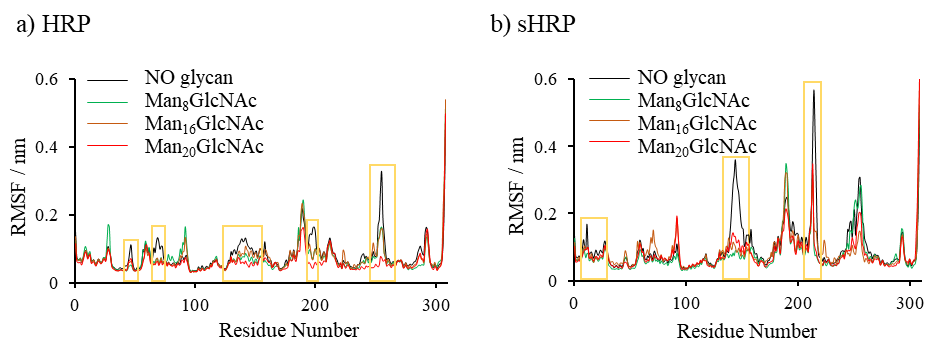


Figure S7. RMSF values of: a) HRP protein and b) sHRP protein. In yellow squares are presented fragments of protein where fluctuations are decreased by introducing glycosylation. Backbone carbon atoms (Cα) of every amino acid from protein backbone was considered in calculation.


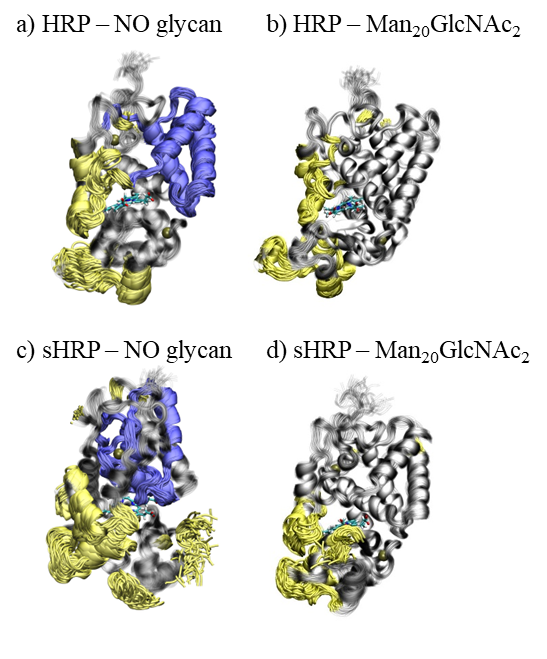


Figure S8. Side view of aligned snapshots of (glyco)protein every 10 ns from trajectory. In yellow are coloured residues which fluctuations decreased the most in glycoprotein Man_20_GlcNAc_2_ (fluctuation decreased by > 0.03 nm). In blue are presented not very flexible amino acids which glycans do not affect. Mentioned fluctuations are on one side of protein. Glycans in glycoprotein (b and d) are not shown for clarity.


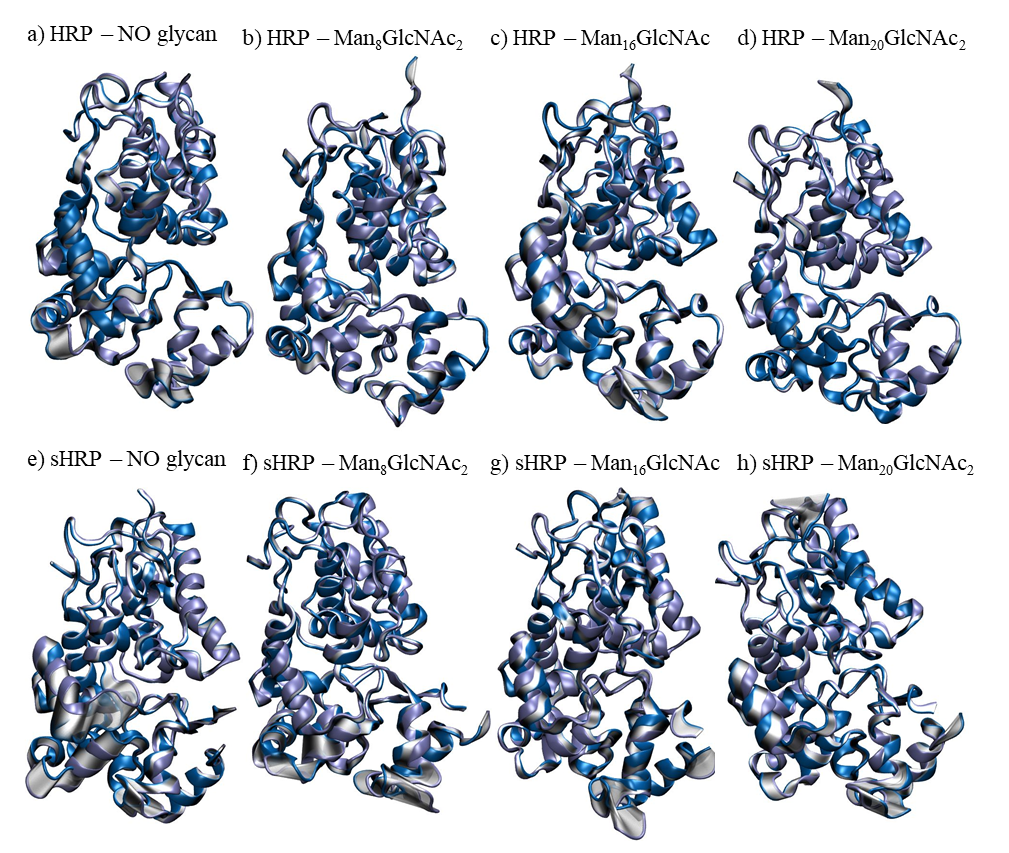


Figure S9. Movements along the first eigenvector PC1 of PCA performed for all proteins separately. Blue and ice blue are two extreme structures, structures in-between are colored gray.


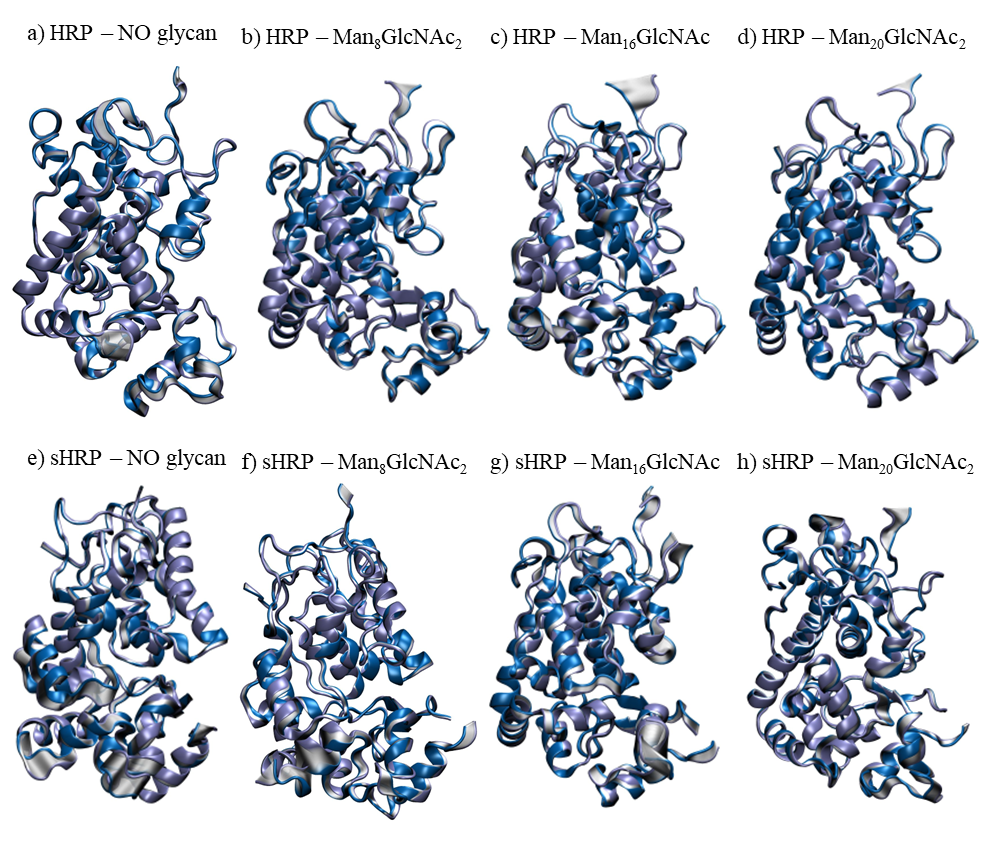


Figure S10. Movements along the second eigenvector (PC2) of PCA performed for all proteins separately. Blue and ice blue are two extreme structures, structures in-between are colored gray.

Table S3: Contribution of PC1-PC5 of PCA performed for each system.

| **HRP** | **NO glycan** | **Man_8_GlcNAc_2_** | **Man_16_GlcNAc_2_** | **Man_20_GlcNAc_2_** |
| --- | --- | --- | --- | --- |
| **PC1** | 9.9 % | 9.0 % | 13.1 % | 5.5 % |
| **PC2** | 6.5 % | 6.3 % | 5.6 % | 5.2 % |
| **PC3** | 4.6 % | 4.1 % | 4.4 % | 3.9 % |
| **PC4** | 3.6 % | 3.6 % | 3.6 % | 3.4 % |
| **PC5** | 2.9 % | 2.8 % | 3.1 % | 2.9 % |
| **sHRP** | **NO glycan** | **Man_8_GlcNAc_2_** | **Man_16_GlcNAc_2_** | **Man_20_GlcNAc_2_** |
| **PC1** | 19.8 % | 17.0 % | 13.4 % | 14.0 % |
| **PC2** | 6.8 % | 6.6 % | 11.4 % | 9.0 % |
| **PC3** | 5.0 % | 3.8 % | 5.0 % | 4.9 % |
| **PC4** | 4.2 % | 3.3 % | 3.6 % | 3.5 % |
| **PC5** | 3.6 % | 3.2 % | 3.0 % | 2.3 % |

Table S4: Average fluctuations and standard deviation of specific regions of protein.

| **HRP** | NO glycan | Man_8_GlcNAc_2_ | Man_16_GlcNAc_2_ | Man_20_GlcNAc_2_ |
| --- | --- | --- | --- | --- |
| Central region **I** / × 10^-1^ nm | 1.1±0.1 | 0.7±0.1 | 0.8±0.1 | 0.6±0.1 |
| Peripheral region **II** / × 10^-1^ nm | 1.4±0.6 | 1.0±0.4 | 1.1±0.4 | 0.7±0.3 |
| **sHRP** | NO glycan | Man_8_GlcNAc_2_ | Man_16_GlcNAc_2_ | Man_20_GlcNAc_2_ |
| Central region **I** / × 10^-1^ nm | 2.1±0.8 | 0.8±0.1 | 0.9±0.1 | 1.1±0.2 |
| Peripheral region **II** / × 10^-1^ nm | 1.8±0.5 | 1.8±0.8 | 1.2±0.7 | 1.3±0.5 |
| Cut-site region **III** / × 10^-1^ nm | 3.8±1.6 | 1.3±1.1 | 1.7±0.7 | 1.4±1.1 |
